# The complete mitochondrial genome of *Barbatula nuda* and *B. toni* (Teleostei: Nemacheilidae)

**DOI:** 10.1080/23802359.2019.1641435

**Published:** 2019-07-16

**Authors:** Yinpen Yang, Hao Chen, Yongxia Chen

**Affiliations:** College of Life Sciences, Hebei University, Baoding, PR China

**Keywords:** *Barbatula nuda*, *Barbatula toni*, mitochondrial genome, Nemacheilidae

## Abstract

In this study, we cloned and sequenced the complete mitochondrial genome of *Barbatula nuda* and *Barbatula toni* with 16,620 and 16,619 nucleotides, respectively. The complete mitogenomes of the two species both include 13 protein-coding genes (PCGs), 2 ribosomal RNA genes, 22 transfer RNA genes, and a non-coding control region. All PCGs of the two species are initiated with ATG or GTG (only for *COI*) and ended with complete (TAA and TAG) or incomplete (TA and T) stop codons. The complete mitochondrial genomes would provide important molecular data for further phylogenetic analyses.

The genus *Barbatula* is small benthic freshwater loaches that are widely distributed in the world from Spain to Korea and Japan (Kottelat and Freyhof 2007). In recent decades, the mitochondrial genomes of two species from Asia have been sequenced and used for phylogenetic analysis (Saitoh et al. 2006; Wang et al. 2016). Saitoh et al. (2006) firstly reported the complete mitochondrial genomes of *B. toni* which was collected from the Ussri River (Genbank accession number is AB242162). Yu et al. (2016) reported another complete mitochondrial genome of *B. toni*, which was collected from the Yeongok River (Genbank accession number is KM405199). The mitochondrial genomes of *B. nuda* were reported by Zhao et al. (2015) and the specimens used were collected from the Irtysh River (Genbank accession number is KF574248). However, according to the current taxonomic research (Chen et al. 2019), the reported species ‘*B. nuda*’ is actually *B. altayensis* and the species ‘*B. toni*’ are two species different from *B. toni*. It is necessary to reextract the whole genome of these two species based on the morphological classification.

Here, we re-report the complete mitogenome of these two species. Specimens were kept in the Hebei University Museum (Baoding City, Hebei Province, China), they are given their accession number 1608147 and 1608497. The sample was immersed in 95％ alcohol and stored in a -21 °C refrigerator. The individuals of *B. nuda* and *B. toni* were collected from the Taizi River at Liaoyang City in Liaoning Province and Yin River at Chifeng City in Inner Mongolia, respectively. All the fish individuals were identified according to the morphological characteristics described by Chen et al. (2019). Genomic DNA was extracted from muscle tissue using a TIANamp® Genomic DNA kit following the manufacturer’s protocol, the kit is originated in Haidian District, Beijing, China, and the manufacturer's headquarters is located in Beijing. A total of 21 primer pairs were designed and used for amplifying the mitochondrial genome sequences.

After DNA sequencing and assembly, the complete mtDNA sequences of *B. nuda* and *B. toni* were obtained and have been deposited in GenBank under the accession number MK900634 and MK900633, respectively. The mitochondrial genome of *B. toni* with 16,620 nucleotides in length consists of 28.27% A, 26.51% T, 27.29% C, and 17.94% G, with an AT content of 54.78%. The mitochondrial genome of *B. nuda* was 16,619 nucleotides in length and consists of 28.18% A, 26.49% T, 27.35% C, and 17.99% G, with an AT content of 54.67%. Both the mitogenomes contain 13 protein-coding genes (PCGs), 2 ribosomal RNA genes (12 S rRNA and 16 S rRNA), 22 transfer RNA genes, and a non-coding control region (D-loop). Most of these genes were encoded on the H-strand, although the ND6 gene and eight tRNA genes were encoded on the L-strand.

Based on the Cyt *b* sequence, the phylogenetic relationship among *Barbatula* species was reconstructed by using the Bayesian inference (BI) and maximum likelihood (ML) methods through MrBayes (Ronquist et al. 2012) and RAxML (Stamatakis 2014), respectively. Both the ML and BI phylogenetic trees showed an identical topology (Figure 1). Within the genus *Barbatula*, fish individuals from *B. nuda* and *B. toni* can be clustered into a strong support, respectively. The specimen of ‘*B. nuda*’ reported by Zhao et al. (2015) were closely related to *B. toni*. Considering the collection of the species they used, only two species have been recorded in Irtysh River drainage, *B. toni* and *B. altayensis* (Li et al. 1966; Zhu 1992; Kottelat 2012; Prokofiev 2016), our molecular data indicates that it is not *B. toni*, it should be the *B*. *altayensis* instead of *B. nuda*. The sequence of ‘*B. toni*’ upload by Saitoh et al. (2006) formed a lineage with another individual which from the same drainage and then closely related to *B. pechiliensis* and *B. zhangwuensis*. The specimen of ‘*B. toni*’ reported by Yu et al. (2016) was closely related to *B. liaoyangensis*. According to the research of the genus *Barbatula* in North-eastern China by Chen et al. (2019), *B. toni* is mainly distributed in Heilongjiang River and West Liao River drainage in north-eastern China, not the Jilin Province and Liaoning Province. Geographically, Korea is close to the Jilin Province and Liaoning Province of China, *B. toni* may be impossible to have a distribution in Korea.

**Figure 1.**
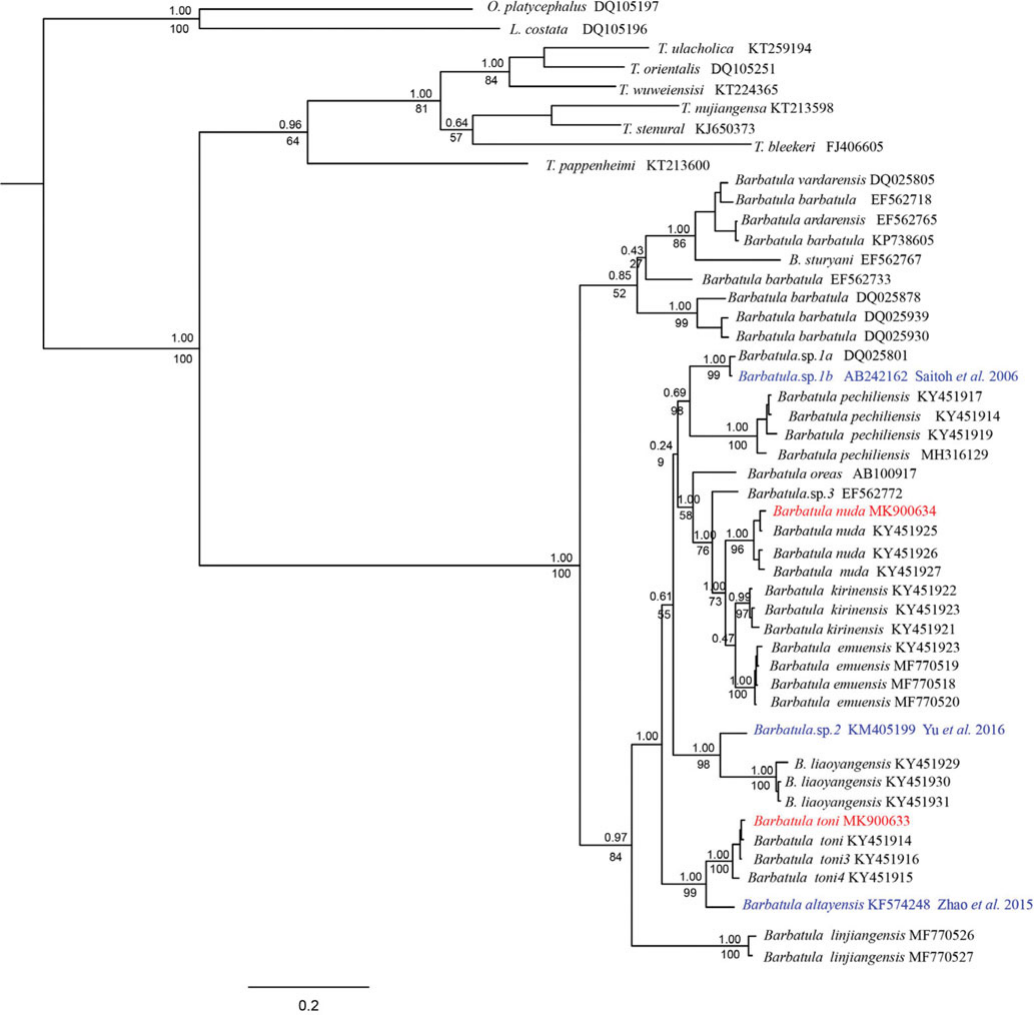
Bayesian tree inferred from mitochondrial Cyt *b* gene sequences. Clade credibility values were given for nodes with bootstrap support for ML (below branch) and posterior probability for Bayesian inferences (above branch).

## References

[CIT0001] Chen H, Zhang H, Chen YX, Jörg F 2019 A review of the *Barbatula* loaches from north-eastern China, with the description of four new species (Teleostei, Nemacheilidae). Zootaxa. 4565:1–036. 10.11646/zootaxa.4565.1.1 31716487

[CIT0002] Kottelat M, Freyhof J 2007 Handbook of European freshwater fishes. Biologia. 52:1–127.

[CIT0003] Kottelat M 2012 *Conspectus cobitidum*: an inventory of the loaches of the world (Teleostei: Cypriniformes: Cobitoidei). Raffles B Zool. 26:1–199.

[CIT0004] Li SZ, Dai DY, Zhang SY 1966 Notes on a collection fishes from north Sinklang, China. Acta Zool Sin. 18:41–56.

[CIT0005] Prokofiev AM 2016 Redescription and systematic position of nominal loach species *Nemacheilus compressirostris* and *N. sibiricus* (Nemacheilidae). J Ichthyol. 56:488–497.

[CIT0006] Ronquist F, Teslenko M, van der Mark P, Ayres DL, Darling A, Höhna S, Larget B, Liu L, Suchard MA, Huelsenbeck JP 2012 MrBayes 3.2: efficient Bayesian phylogenetic inference and model choice across a large model space. Syst Biol. 61:539–542. 22357727 10.1093/sysbio/sys029 PMC3329765

[CIT0007] Saitoh K, Sado T, Mayden RL, Hanzawa N, Nakamura K, Nishida M, Miya M 2006 Mitogenomic evolution and interrelationships of the Cypriniformes (Actinopterygii: Ostariophysi): the first evidence toward resolution of higher-level relationships of the world’s largest freshwater fish clade based on 59 whole mitogenome sequences. J Mol Evol. 63:826–841. 17086453 10.1007/s00239-005-0293-y

[CIT0008] Stamatakis A 2014 RAxML version 8: a tool for phylogenetic analysis and post-analysis of large phylogenies. Bioinformatics. 30:1312–1313. 24451623 10.1093/bioinformatics/btu033 PMC3998144

[CIT0009] Wang Y, Shen YJ, Feng CG, Zhao K, Song ZB, Zhang YP, Yang LD, He SP 2016 Mitogenomic perspectives on the origin of Tibetan loaches and their adaptation to high altitude. Sci Rep. 6:29690. 27417983 10.1038/srep29690 PMC4945904

[CIT0010] Yu J-N, Jun J, Lim CE, Kim S 2016 Sequence and organization of the complete mitogenome of a Siberian stone loach, *Barbatula toni* (Dybowsky, 1869) (Cypriniformes: Balitoridae). Mitochondrial DNA. 27:1798–1799. 26305807 10.3109/19401736.2014.963818

[CIT0011] Zhao X, Hu SF, Xie P, Ao MJ, Cai LG, NIU JG, Ma XF 2015 The complete mitochondrial genome of *Barbatula nuda* (Cypriniformes: Nemacheilidae). Mitochondrial DNA. 26:692–693. 24102606 10.3109/19401736.2013.840611

[CIT0012] Zhu SQ 1992 Three new species of Nemacheilinae fishes from China (Cypriniformes: Cobitidae). Acta Zootaxon Sin. 17:241–247.

